# Predictors of Successful Tobacco Cessation After Receiving an E-Cigarette Based Smoking Cessation Intervention

**DOI:** 10.1177/1179173X241283470

**Published:** 2024-10-30

**Authors:** Ian Pope, Allan Clark, Lucy Clark, Emma Ward, Susan Stirling, Pippa Belderson, Caitlin Notley

**Affiliations:** Norwich Medical School, 6106 University of East Anglia, Norwich, UK

**Keywords:** Smoking cessation, predictors, emergency medicine, electronic cigarettes

## Abstract

**Introduction:**

E-cigarettes have been shown to be effective for tobacco smoking cessation. Predicting those who are most likely to achieve smoking abstinence after receiving an e-cigarette based smoking cessation intervention could help to target interventions more efficiently.

**Methods:**

A secondary analysis of baseline characteristics of 505 people who received an emergency department based smoking cessation intervention incorporating brief advice, provision of an e-cigarette starter kit and referral to stop smoking services. Gender, ethnicity, age, employment status, deprivation, partner smoking status, cigarettes per day, motivation to quit, cigarette dependence and previous e-cigarette use were assessed as predictors of abstinence. Self-reported smoking status was collected 6 months after intervention delivery.

**Results:**

At 6 months 169/505 (33%) of those who received the intervention self-reported abstinence. The groups that were more likely to report having quit were females (37.4% of females vs 31.0% of males), older people (41.1% of over 50s vs 33.3% of under 35s), lighter smokers (36.4% of those who smoked less than 10 cigarettes per day vs 30.7% for those who smoked over 20) and more motivated quitters (35.6% for those with high motivation vs 29.2% for those with low motivation). However, in multiple logistic regression, when adjusting for the other factors, no factors significantly predicted smoking abstinence. Degree of nicotine dependence was very similar between those who quit and those who did not.

**Conclusion:**

The study found no baseline factors that could predict successful smoking cessation with e-cigarettes. Consequently, this study does not support the use of a targeted e-cigarette-based smoking cessation intervention, suggesting the adoption of a more universal approach.

## Introduction

E-cigarettes and behavioural support have been shown to be effective for smoking cessation.^1,2^ Predicting who would be most likely to quit after receiving an e-cigarette smoking cessation intervention has the potential to allow targeting of the intervention at those with the highest chance of success and therefore improving intervention cost-effectiveness. However, consideration also needs to be made of those who have the most to gain from successfully quitting and the impact on health inequalities.

It is known that lower levels of cigarette dependence increases the chance of a successful quit attempt at the population level.^3^ There is mixed evidence regarding socio-demographic variables with some indication that higher social grade may increase the success of a quit attempt and older people may be more likely to quit. There is some evidence that late initiation of cigarette smoking, longer duration of previous quit attempts, lack of depression or anxiety, low to moderate nicotine dependence, absence of alcohol problems, sustained levels of motivation, being married and not having any smokers in the household are associated with smoking cessation.^4-13^ However very little is known about predictors after receiving an e-cigarette intervention. One survey of 889 Canadian adults who had tried to quit smoking using an e-cigarette found that a positive experience of vaping was the most important predictor of perceived success in vaping-assisted smoking cessation with the other factors being younger age, having vaped 100 times and vaping shortly after waking up.^9^

The aim of this study is to examine factors predicting successful tobacco cessation after receiving an e-cigarette based smoking cessation intervention.

## Method

The Cessation of Smoking Trial in the Emergency Department (COSTED) was a multi-centre randomised controlled trial which recruited adults (aged 18 years or older) who reported smoking tobacco daily, attending one of six EDs for medical treatment or accompanying someone attending for medical treatment. Participants were screened while they were in the ED. People were excluded if they had an expired carbon monoxide (CO) of <8 parts per million (ppm), required immediate medical treatment, were in police custody, had a known allergy to nicotine, were current dual users (defined as daily e-cigarette use), were considered not to have capacity to consent or had already taken part in the trial. Further details including the power calculation can be found in the published protocol and results.^14,15^ Participants randomised to the intervention received (1) brief theory driven^16^ smoking cessation advice (up to 15 mins), (2) the provision of an e-cigarette starter kit and 11 pods plus instruction in its use (up to 15 mins) and (3) referral to local stop smoking services.

Participants completed baseline measures prior to being randomised into the trial. These included demographic variables, smoking behaviour, motivation to stop smoking scale^17^ and Fagerstrom test for nicotine dependence.^18^

Self-reported smoking status was assessed at 6 months by asking “Are you currently smoke free? By smoke free we mean not smoking any form of tobacco (cigarettes, roll-ups, cigarillos, pipes). It does not include e-cigarettes.”^19^ Those who responded Yes (regardless of number of lapses in the previous 6 months) were counted as abstinent. Those who did not respond were assumed still to be smoking.

All participants in the intervention arm were included in this secondary analysis. Predictors of quitting were investigated using logistic regression analysis. Firstly, univariate odds ratios were calculated for each of the ten independent variables (1 continuous, 5 binary and 4 categorical) individually. In order to control for potential confounding effects, all independent variables were entered into a multivariable logistic regression. The results of the analyses are shown as self-reported quitting vs not quitting at 6-months from randomisation. All data were analysed in Stata 18.0 and the level of significance was set at 5% with 95% confidence intervals reported.

## Results

505 participants were randomised to receive the intervention. Full details of participant flow and follow-up rates can be found in the published results.^15^ At 6 months 169/505 (33%) of those who received the intervention self-reported abstinence.

Table 1 shows the quit rate by demographic and smoking variables, the quit rate was higher in those who were female, white British, older, unemployed, smoked less than 10 cigarettes per day and had higher motivation to quit. However, Table 2 shows the results of the multivariable logistic regression. The adjusted odds of smoking abstinence were not significantly different for any factors. There was some indication that those over 50 were more likely to quit however this was not significant (OR = 1.58, 95% CI = 0.97-2.57, *P* = .065).

**Table 1.** Quit Rate by Demographic and Smoking Variable.

		Quit (n = 169) 33.5%	Did Not Quit (n = 336) 66.5%
Gender	Male	96/310 (30.97)	214/310 (69.03)
	Female	73/195 (37.44)	122/195 (62.56)
Ethnicity	White British	126/369 (34.15)	243/369 (65.85)
	White other	21/69 (30.43)	48/69 (69.57)
	Asian	8/32 (25.0)	24/32 (75.00)
	Black	11/28 (39.29)	17/28 (60.71)
	Other	3/7 (42.86)	4/7 (57.14)
Age	18-35	69/207 (33.33)	138/207 (66.67)
	36-50	49/174 (28.16)	125/174 (71.84)
	>50	51/124 (41.13)	73/124 (58.87)
Employment status	Employed	46/147 (31.29)	101/147 (68.71)
	Unemployed	123/358 (34.36)	235/358 (65.64)
Deprivation quintile	1 (least deprived)	48/153 (31.37)	105/153 (68.63)
	2	51/150 (34.00)	99/150 (66.00)
	3	29/91 (31.87)	62/91 (68.13)
	4	26/64 (40.62)	38/64 (59.38)
	5 (most deprived)	15/47 (31.91)	32/47 (68.09)
Partner smoking status	Live with or partner smoker	77/229 (33.62)	152/229 (66.38)
	Do not live with or have partner that smokes	92/276 (33.33)	184/276 (66.67)
Cigarettes per day	≤10	59/162 (36.42)	103/162 (63.58)
	11-19 per day	51/151 (33.77)	100/151 (66.23)
	≥20	59/192 (30.73)	133/192 (69.27)
Motivation to quit	High	120/337 (35.61)	217/337 (64.39)
	Low	49/168 (29.17)	119/168 (70.83)
Fagerstrom score (dependency) (0-10), mean (SD)		4.96 (2.34)	4.91 (2.15)
Previous use of an e-cigarette	Yes	50/141 (35.46)	91/141 (64.54)
	No	119/364 (32.69)	245/364 (67.31)

**Table 2.** Multivariate Logistic Model Predicting Quitting.

		Adjusted Odds Ratio	95% CI	*P*-value
Gender	Male	1.00 (reference)		
	Female		1.39 (0.93,2.08)	0.112
Ethnicity	White British	1.00 (reference)		
	White other		0.82 (0.46,1.46)	0.502
	Asian		0.6 (0.25,1.44)	0.251
	Black		1.39 (0.6,3.24)	0.441
	Other		1.7 (0.35,8.22)	0.506
Age	<36	1.00 (reference)		
	36-50		0.8 (0.51,1.27)	0.344
	>50		1.58 (0.97,2.57)	0.068
Employment status	Employed	1.00 (reference)		
	Unemployed		1.16 (0.75,1.81)	0.503
Deprivation quintile	1	1.00 (reference)		
	2		1.15 (0.7,1.9)	0.581
	3		1.08 (0.6,1.92)	0.805
	4		1.42 (0.76,2.66)	0.277
	5		0.99 (0.48,2.03)	0.969
Living with someone or having a partner who smokes	No	1.00 (reference)		
	Yes		1.17 (0.79,1.75)	0.426
Cigarettes per day	≤10	1.00 (reference)		
	11-19 per day		0.81 (0.48,1.36)	0.421
	≥20		0.7 (0.39,1.27)	0.238
Motivation to quit	High	1.00 (reference)		
	Low		1.41 (0.93,2.14)	0.104
Fagerstrom score (dependency)			1.04 (0.93,1.15)	0.514
Previous e-cigarette use	No	1.00 (reference)		
	Yes		0.85 (0.55,1.3)	0.45
		No previous E-cigarette use	119/364 (32.69)	245/364 (67.31)

## Discussion

This study did not find any baseline factors that significantly predicted self-reported smoking abstinence at 6-months, when controlling for other variables.

In keeping with previous evidence there were higher quit rates amongst those who were female, older, lighter smokers and more motivated to quit. However these difference were not significant in the adjusted model.

In contrast to previous studies level of dependency was very similar between those who quit and those who did not. This may be because using an e-cigarette continues to meet the need for nicotine and therefore dependency may not predict quitting success as has been shown to be the case in quit attempts not involving e-cigarettes. This is a potentially important finding given the previous assumption that dependence is the most powerful predictor of success. This aligns with the existing evidence around the existence of “accidental quitters” i.e., people who start using an e-cigarette with no intention of quitting tobacco but end up quitting.^20^

Also in contrast to previous research there was no significant difference in quit rates between different levels of deprivation, if this finding is found to be consistent across other studies this raises the prospect of e-cigarette based smoking cessation interventions potentially offering a route to combat health inequalities driven by differential smoking rates.

The strengths of this study are the relatively large sample size and quit rate and the fact that participants were opportunistically recruited from the emergency department therefore represent a diverse group in terms of demographics and motivation to quit.

Weaknesses of this study are that some participants were lost to follow-up and were assumed not to be abstinent which may be a conservative assumption. The fact participants were recruited in the emergency department may limit the generalisability of the findings to other settings. The study was not powered to look for any of the associations presented therefore any conclusions must be interpreted in this light. The trial used a specific e-cigarette and behaviour change approach and it is possible that an alternative device or approach may have resulted in different results. The predictors not being statistically significant in the adjusted model may be due to underlying biases or confounding effects.

## Conclusions

The study found no baseline factors that could predict successful smoking cessation with e-cigarettes. Consequently, this study does not support the use of a targeted e-cigarette-based smoking cessation intervention, suggesting the adoption of a more universal approach.
